# Supplementary figures and images for: Cell elimination strategies upon identity switch via modulation of apterous in Drosophila wing disc
Source: PLoS Genet. 2019 Dec 26;15(12):e1008573. doi: 10.1371/journal.pgen.1008573 (PMC6952109; doi:10.1371/journal.pgen.1008573)

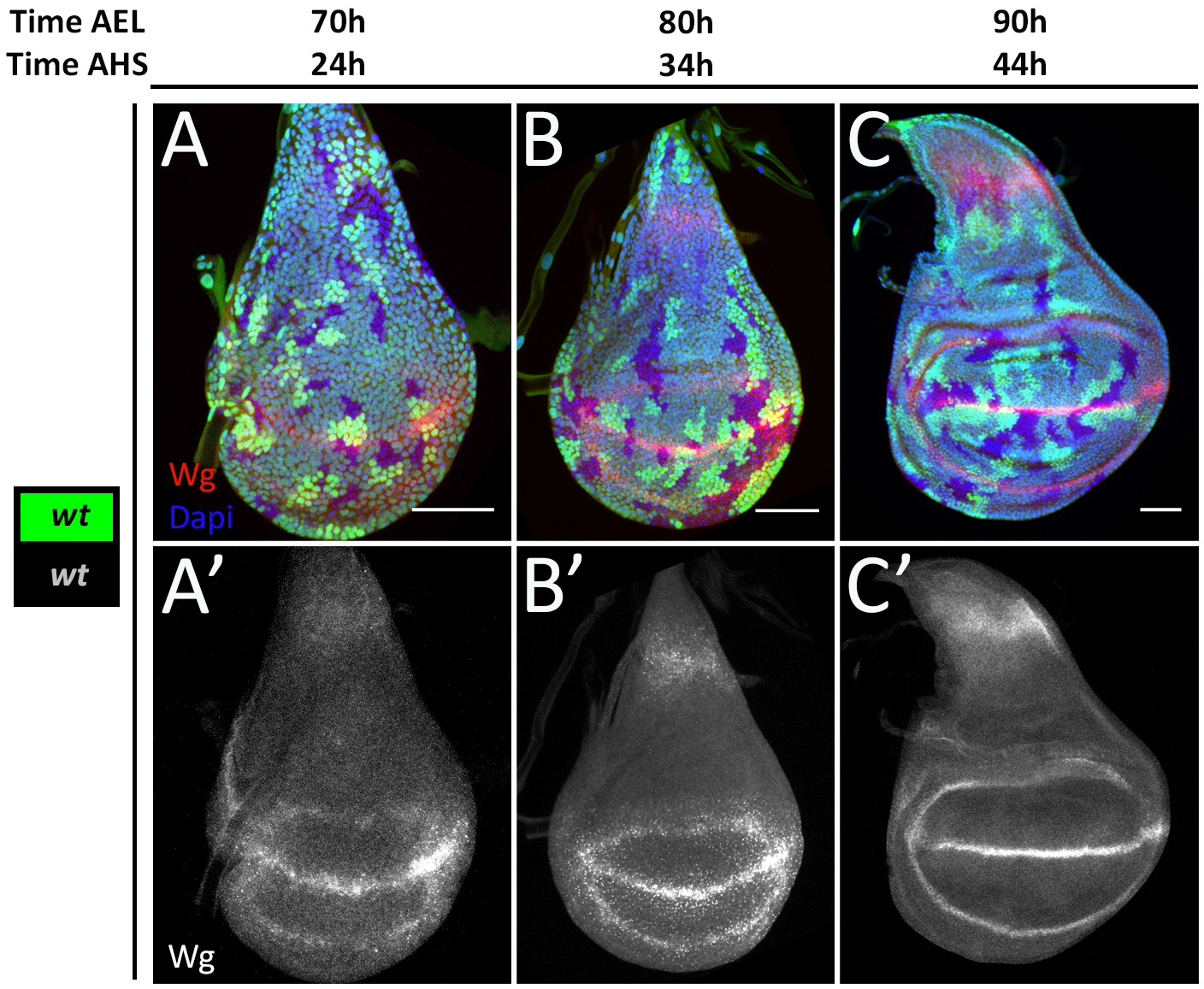

Supplement: S1 Fig — (A-C) Wing discs of the indicated times containing wild-type sister clones that are marked by either 2 copies of GFP or absence of GFP. (A’-C’) Wg channel of A-C. Quantifications of the remained, relocated and eliminated wild-type clones are shown on the Fig 2G–2I, blue lines. Scale bars represent 50μm. (TIF) [file pgen.1008573.s001.tif]

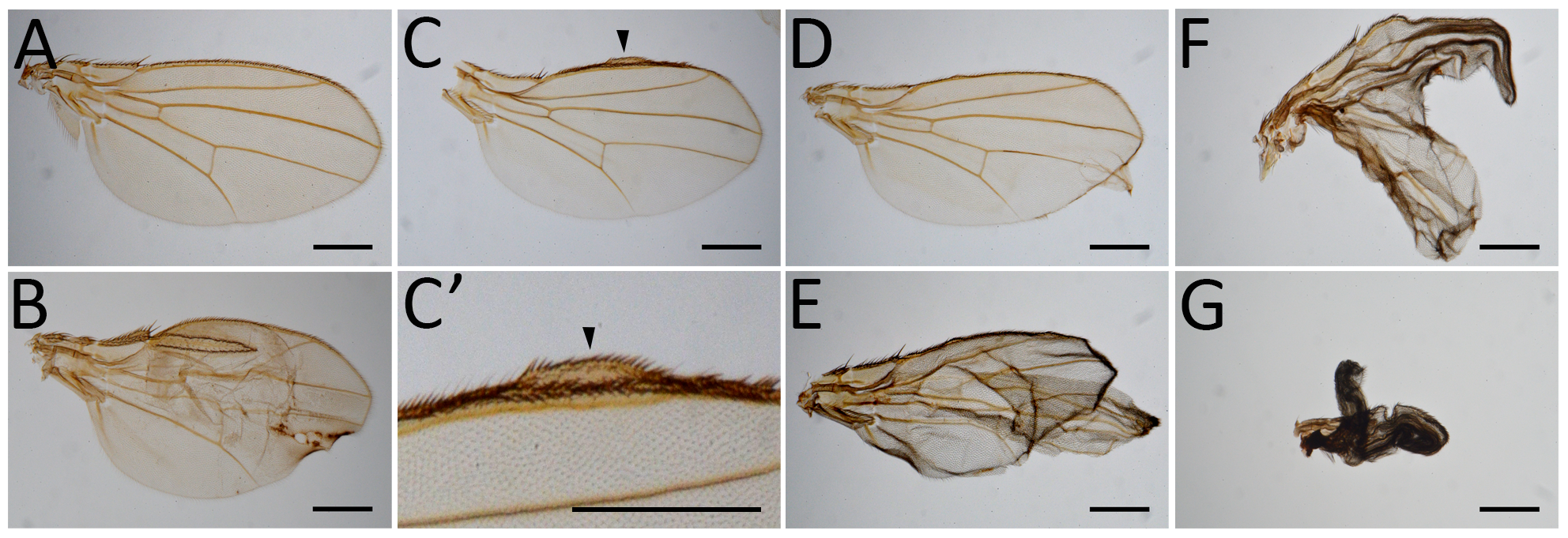

Supplement: S2 Fig — (A) Wild-type wing. (B-G) Wings after induction of apDG8 clones during second instar contain different deformations: ectopic margin formation (B); wing margin duplication (C-C’, arrowheads); blister-like outgrowths (D-E); and wing duplications (F-G). Scale bars represent 500μm. (TIF) [file pgen.1008573.s002.tif]

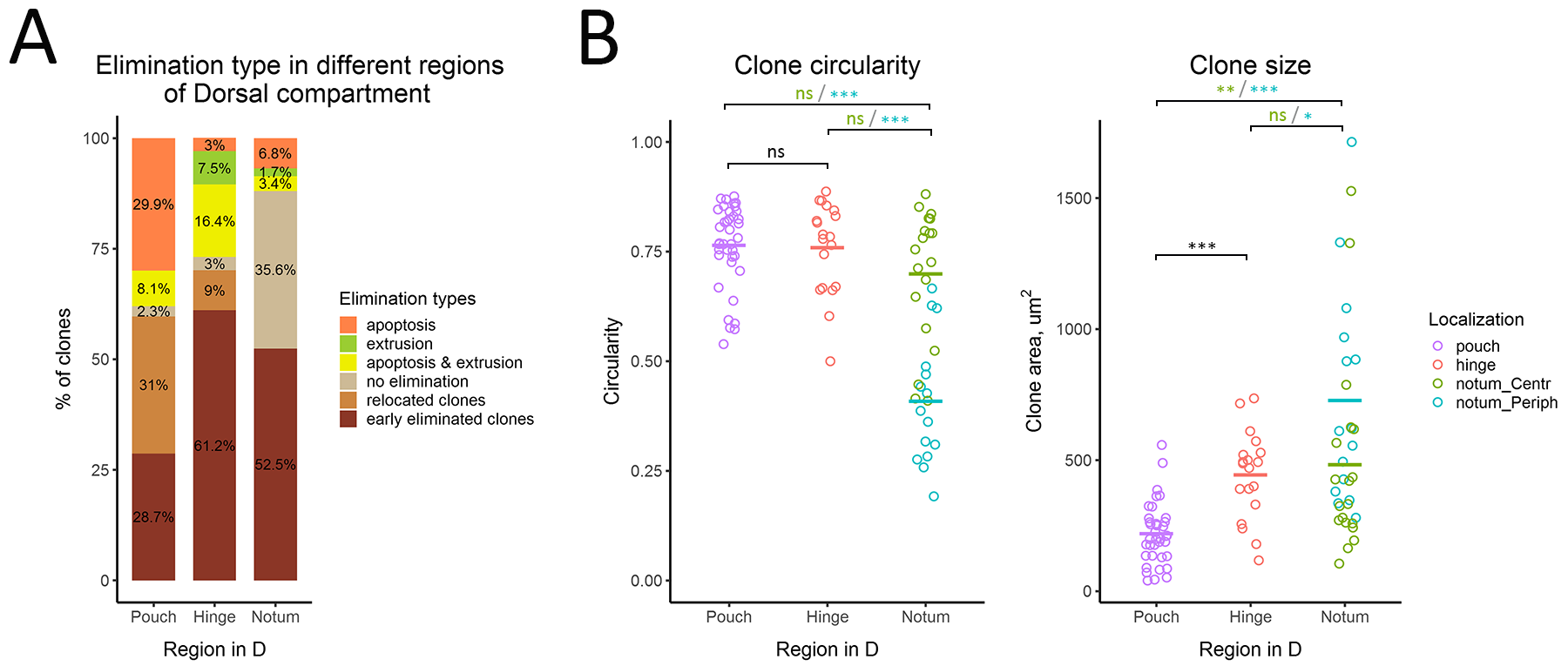

Supplement: S3 Fig — (A) Quantification of dorsal mutant clones in different regions depending on the evidence of elimination type including clones that have been eliminated (early eliminated) or relocated by the time of analysis. The analysis was based on quantification of wt clones and their mutant sister clones. A total of 213 dorsal wt clones from 23 discs were analyzed: 87 clones were in the pouch, 67 in the hinge and 59 in the notum. (B) The circularity and size of mutant clones remaining in different regions of the dorsal compartment at 50h AHS. The clones in the notum were grouped into 2 categories depending on whether they touch the sides of the disc (notum_Periph) or are in the central part (notum_Centr). Altogether 91 clones were measured. (TIF) [file pgen.1008573.s003.tif]

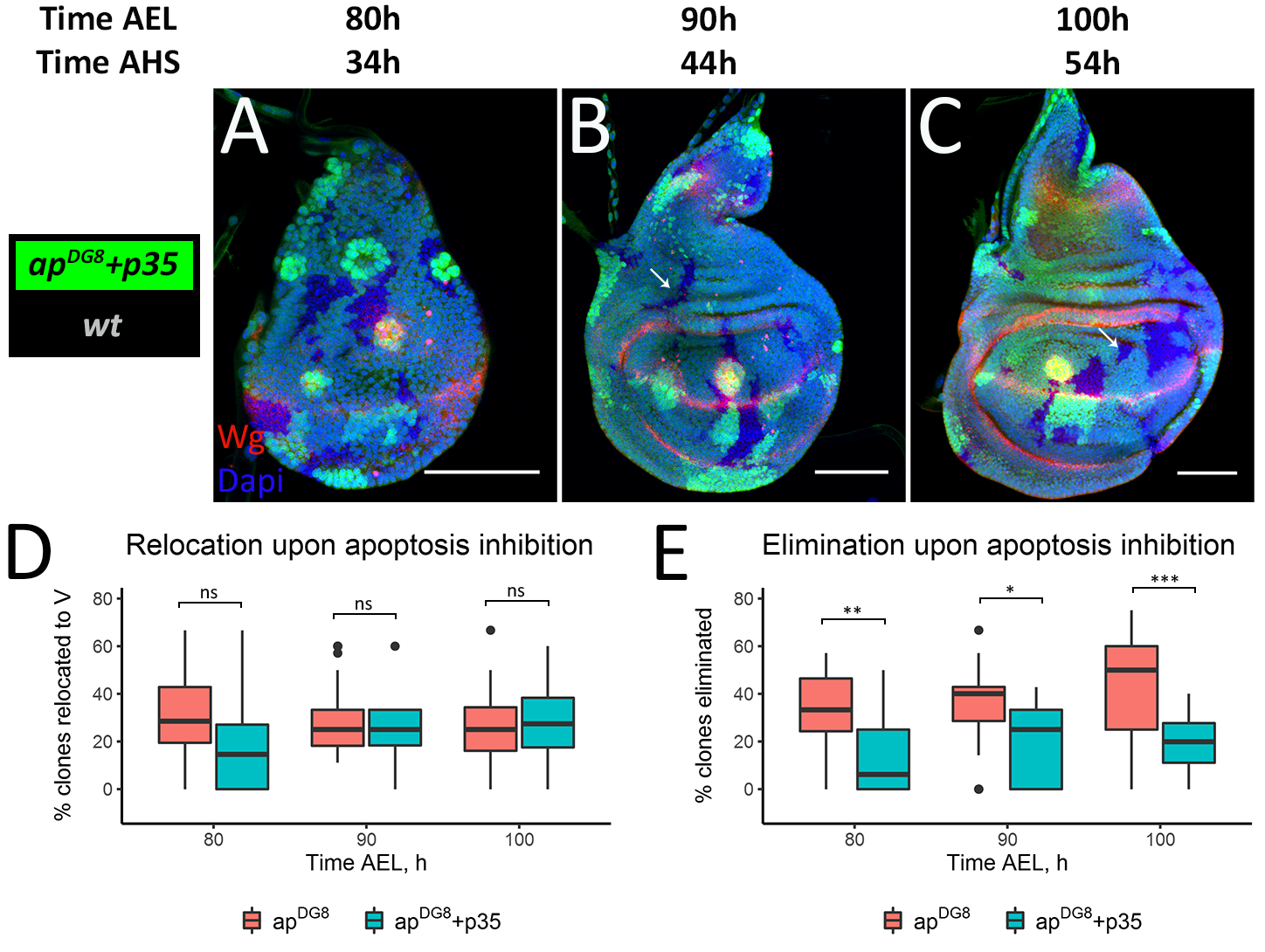

Supplement: S4 Fig — (A-C) Wing imaginal discs of indicated times with apDG8 clones expressing p35 (marked by two copies of GFP) and wild-type sister clones (marked by the absence of GFP). Arrows point to wild-type clones that lost their mutant sisters; (D-E) Comparison of the amount of apDG8 clones (data from the Fig 2) with the amount of apDG8 + p35 clones that were relocated to the ventral compartment (D) or completely eliminated (E). At least 15 discs with apDG8 clones and 12 discs with apDG8 + p35 clones were analyzed. Scale bars represent 50μm. (TIF) [file pgen.1008573.s004.tif]

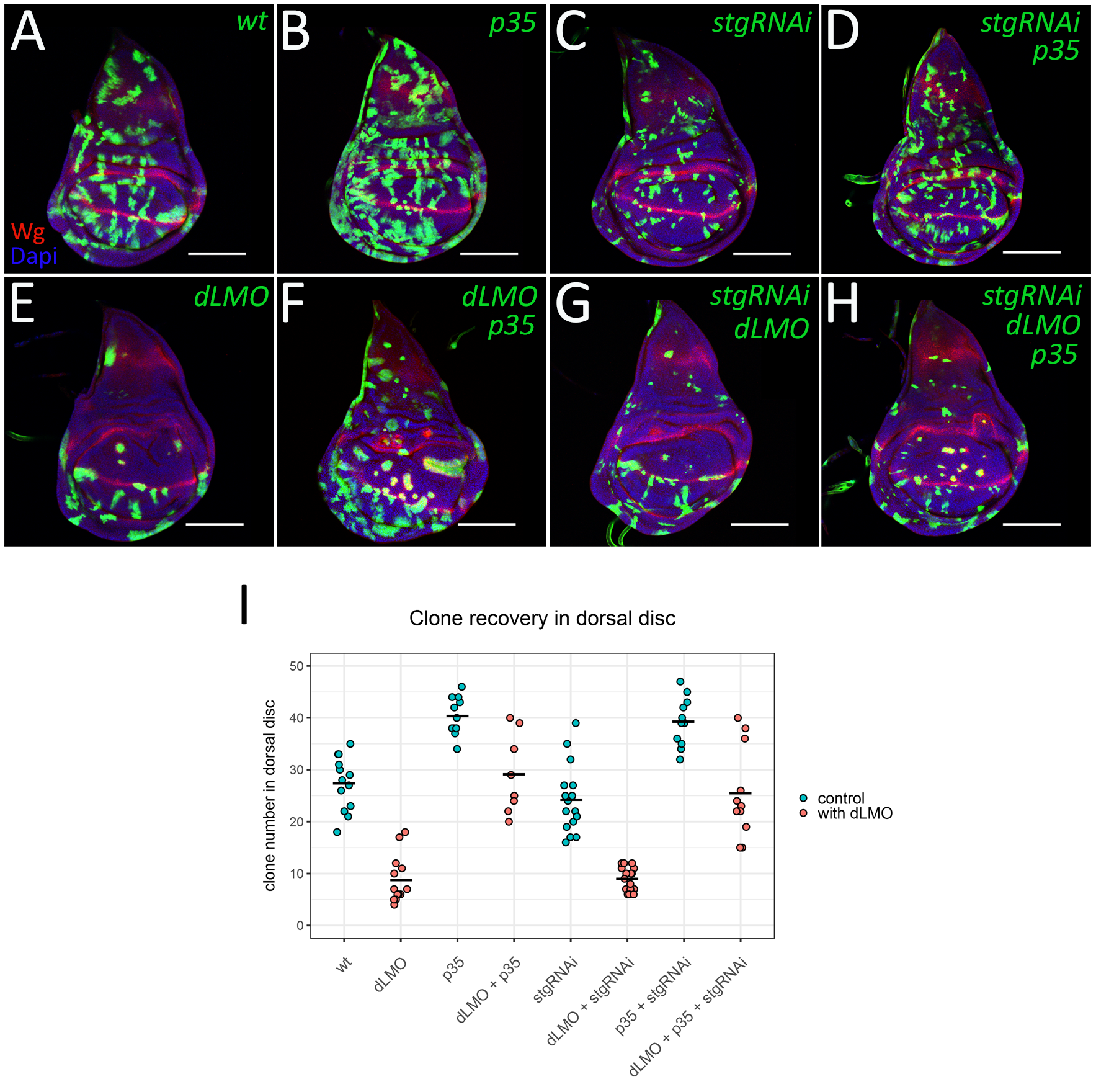

Supplement: S5 Fig — (A-H) Third instar wing discs containing wild-type (A), p35 (B), stgRNAi (C), p35+stgRNAi (D), dLMO (E), dLMO+p35 (F), stgRNAi+dLMO (G) and stgRNAi+dLMO+p35 (H) clones. (I) Clone recovery rate in dorsal compartment for each genotype. Scale bars represent 100μm. (TIF) [file pgen.1008573.s005.tif]

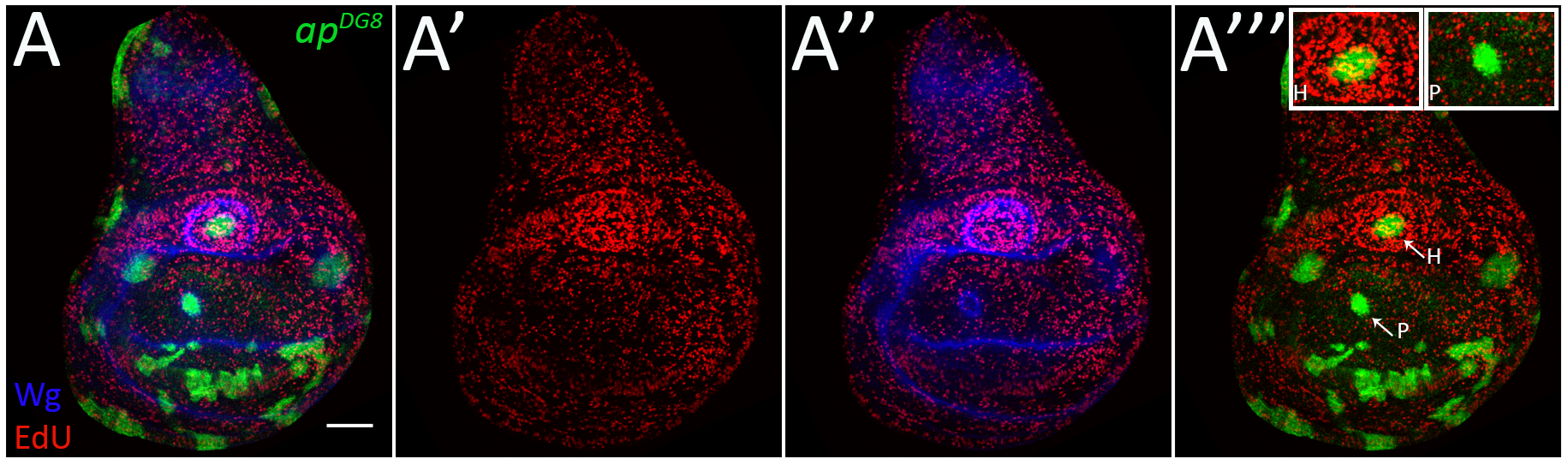

Supplement: S6 Fig — EdU cell proliferation assay of the third instar wing disc containing apDG8 clones. (A) Merged image (apDG8 clones, EdU and Wg staining). (A’) EdU channel alone. (A”) EdU and Wg channels. (A”’) apDG8 clones and EdU staining. The insets show enlarged images of single clones from dorsal pouch (P) and dorsal hinge (H). Scale bar represents 50μm. (TIF) [file pgen.1008573.s006.tif]
