# Supplementary material for: Cell elimination strategies upon identity switch via modulation of apterous in Drosophila wing disc
Source: PLoS Genet. 2019 Dec 26;15(12):e1008573. doi: 10.1371/journal.pgen.1008573 (PMC6952109; doi:10.1371/journal.pgen.1008573)
Supplement: S1 Table — Detailed genotypes and experimental conditions of data represented in the figures. (DOCX) [file pgen.1008573.s007.docx]

| **Figure** | **Genotype** | **Time AEL, h** | | | **Heat-shock duration, min** |
| --- | --- | --- | --- | --- | --- |
|  |  | **Egg collection** | **Heat-shock** | **Dissection** |  |
| 1A | *yw hsflp /yw; FRT^f00878^/ FRT^f00878^ tub-Gal80; tub-Gal4 UAS-GFP/+* | 4 | 48-52 | 110-114 | 30 |
| 1B | *yw hsflp /yw; FRT^f00878^ ap^DG8^/ FRT^f00878^ tub-Gal80; tub-Gal4 UAS-GFP / +* | 4 | 48-52 | 110-114 | 30 |
| 1C | *yw hsflp / w; UAS-Ap / +; act>CD2>Gal4 UAS-GFP / +* | 4 | 46-50 | 100-104 | 12 |
| 2 B | *yw hsflp/(y)w; FRT^f00878^ ap^DG8^ ubi-GFP / FRT^f00878^* | 4 | 42-46 | 86-90 | 30 |
| 2C | *yw hsflp/(y)w; FRT^f00878^ ap^DG8^ ubi-GFP / FRT^f00878^* | 4 | 42-46 | 66-70 | 30 |
| 2D | *yw hsflp/(y)w; FRT^f00878^ ap^DG8^ ubi-GFP / FRT^f00878^* | 4 | 42-46 | 76-80 | 30 |
| 2E | *yw hsflp/(y)w; FRT^f00878^ ap^DG8^ ubi-GFP / FRT^f00878^* | 4 | 42-46 | 86-90 | 30 |
| 2F | *yw hsflp/(y)w; FRT^f00878^ ap^DG8^ ubi-GFP / FRT^f00878^* | 4 | 42-46 | 96-100 | 30 |
| 3B | *yw hsflp/(y)w; FRT^f00878^ ap^DG8^ ubi-GFP / FRT^f00878^* | 4 | 62-66 | 96-100 | 30 |
| 3C | *yw hsflp/(y)w; FRT^f00878^ ap^DG8^ ubi-GFP / FRT^f00878^* | 4 | 62-66 | 106-110 | 30 |
| 4A | *yw hsflp/(y)w; FRT^f00878^ ap^DG8^ ubi-GFP / FRT^f00878^* | 4 | 42-46 | 86-90 | 30 |
| 4B | *yw hsflp / w; UAS-Ap / +; act>CD2>Gal4 UAS-GFP / +* | 4 | 42-46 | 86-90 | 12 |
| 4C | *yw hsflp /yw; FRT^f00878^ ap^DG8^/ FRT^f00878^ tub-Gal80; tub-Gal4 UAS-GFP / +* | 24 | 32-56 | 76-100 | 30 |
| 4D | *yw hsflp / w; UAS-Ap / +; act>CD2>Gal4 UAS-GFP / +* | 4 | 42-46 | 86-90 | 12 |
| 4E | *yw hsflp /yw; FRT^f00878^ ap^DG8^/ FRT^f00878^ tub-Gal80; tub-Gal4 UAS-GFP / +* | 8 | 51-59 | 92-100 | 30 |
| 4F | *yw hsflp/(y)w; FRT^f00878^ ap^DG8^ ubi-GFP / FRT^f00878^* | 8 | 40-48 | 92-100 | 30 |
| 5A | *yw hsflp /yw; FRT^f00878^/ FRT^f00878^ tub-Gal80; tub-Gal4 UAS-GFP/+* | 24 | 32-56 | 76-100 | 30 |
| 5B | *yw hsflp /yw; FRT^f00878^ / FRT^f00878^ tub-Gal80; tub-Gal4 UAS-GFP/ UAS-p35* | 24 | 32-56 | 76-100 | 30 |
| 5C | *yw hsflp /yw; FRT^f00878^ ap^DG8^/ FRT^f00878^ tub-Gal80; tub-Gal4 UAS-GFP/+* | 24 | 32-56 | 76-100 | 30 |
| 5D | *yw hsflp /yw; FRT^f00878^ ap^DG8^ / FRT^f00878^ tub-Gal80; tub-Gal4 UAS-GFP/ UAS-p35* | 24 | 32-56 | 76-100 | 30 |
| 5G | *yw hsflp /yw; FRT^f00878^ ap^DG8^ / FRT^f00878^ tub-Gal80; tub-Gal4 UAS-GFP/ UAS-p35* | 24 | 24-48 | 96-120 | 30 |
| 6A | *yw hsflp / w; UAS-dLMO / IF or CyO; act>CD2>Gal4 UAS-GFP / MKRS* | 8 | 60-68 | 106-114 | 13 |
| 6B | *yw hsflp / hsflp; UAS-dLMO / CyO or IF; act>CD2>Gal4 UAS-GFP / UAS-p35* | 8 | 60-68 | 106-114 | 13 |
| 6C | *yw hsflp / w; UAS-dLMO / UAS-stg-RNAi; act>CD2>Gal4 UAS-GFP / +* | 8 | 60-68 | 106-114 | 13 |
| 6D | *yw hsflp / yw hsflp; UAS-dLMO / UAS-stg-RNAi; act>CD2>Gal4 UAS-GFP / UAS-p35* | 8 | 60-68 | 106-114 | 13 |
| 6F | Same as in 6B | | | | |
| 6G | Same as in 6D | | | | |
| S1A | *yw hsflp/(y)w; FRT^f00878^ ubi-GFP / FRT^f00878^* | 4 | 42-46 | 66-70 | 30 |
| S1B | *yw hsflp/(y)w; FRT^f00878^ ubi-GFP / FRT^f00878^* | 4 | 42-46 | 76-80 | 30 |
| S1C | *yw hsflp/(y)w; FRT^f00878^ ubi-GFP / FRT^f00878^* | 4 | 42-46 | 86-90 | 30 |
| S2A | *yw hsflp / (y)w; FRT^f00878^ / FRT^f00878^ tub-Gal80; tub-Gal4 UAS-GFP / +* | 20 | 46-66 | - | 30 |
| S2B-G | *yw hsflp / (y)w; FRT^f00878^ ap^DG8^ / FRT^f00878^ tub-Gal80; tub-Gal4 UAS-GFP / +* | 20 | 46-66 | - | 30 |
| S3A | *(w) hsflp / (y)w (hsflp); FRT^f00878^ ap^DG8^ ubi-GFP / FRT^f00878^ tub-Gal80; tub-Gal4 UAS-mCherry/ UAS-p35* | 4 | 42-46 | 76-80 | 30 |
| S3B | *(w) hsflp / (y)w (hsflp); FRT^f00878^ ap^DG8^ ubi-GFP / FRT^f00878^ tub-Gal80; tub-Gal4 UAS-mCherry/ UAS-p35* | 4 | 42-46 | 86-90 | 30 |
| S3C | *(w) hsflp / (y)w (hsflp); FRT^f00878^ ap^DG8^ ubi-GFP / FRT^f00878^ tub-Gal80; tub-Gal4 UAS-mCherry/ UAS-p35* | 4 | 42-46 | 96-100 | 30 |
| S4A | *yw hsflp / w; IF or CyO / +; act>CD2>Gal4 UAS-GFP / MKRS* | 8 | 60-68 | 106-114 | 13 |
| S4B | *yw hsflp / hsflp; IF or CyO / +; act>CD2>Gal4 UAS-GFP / UAS-p35* | 8 | 60-68 | 106-114 | 13 |
| S4C | *yw hsflp / w; UAS-stgRNAi / +; act>CD2>Gal4 UAS-GFP / +* | 8 | 60-68 | 106-114 | 13 |
| S4D | *yw hsflp / yw hsflp; UAS-stgRNAi / +; act>CD2>Gal4 UAS-GFP / UAS-p35* | 8 | 60-68 | 106-114 | 13 |
| S4E | Same as in 6A | | | | |
| S4F | Same as in 6B | | | | |
| S4G | Same as in 6C | | | | |
| S4H | Same as in 6D | | | | |
| S5 | *yw hsflp /yw; FRT^f00878^ ap^DG8^/ FRT^f00878^ tub-Gal80; tub-Gal4 UAS-GFP / +* | 6 | 61-67 | 108-114 | 30 |
